# Data based investigation of the peer education methods on self-efficacy in patients with myocardial infarction using a randomized control trial design

**DOI:** 10.1016/j.dib.2018.08.190

**Published:** 2018-09-05

**Authors:** Reza Mohammadpourhodki, Maryam Keramati, Ali Abbasi, Mohammad Hasan Basirinezhad, Aria Dianatinasab, Mostafa Dianatinasab

**Affiliations:** a Department of Nursing, School of Nursing and Midwifery, Shahroud University of Medical Sciences, Shahroud, Iran; b School of Medicine, Mashhad University of Medical Sciences, Mashhad, Iran; c Department of Epidemiology and Biostatistics, School of Public Health, Tehran University of Medical Sciences, Tehran, Iran; d Department of Biochemistry, Student Research Committee, Shiraz University of Medical Sciences, Shiraz, Iran; e Center for Health Related Social and Behavioral Sciences Research, Shahroud University of Medical Sciences, Shahroud, Iran

## Abstract

This database aims to show the effects of peer-education (PE) on self-efficacy in patients with MI referring to Zabol Emam Ali Hospital in 2016.

The data provided in this paper are for a descriptive-analytical and experimental study which included 70 patients with MI that randomly assigned to PE group (*n* = 35) and control group (*n* = 35). We used two tools for data collection in this data article. Patient׳s demographic data questionnaire, consisting of two parts: the first section had questions about general details such as age, education, marital status, while the second section had questions about health status information. Cardiac self-efficacy questionnaire was the second questionnaire. Finally, the data of 30 intervention and 30 control individuals were then analyzed by SPSS software and *P value < 0.05* was considered to be statistically significant. The data indicated that PE can be useful for providing a better care in MI patients, thus, it is recommended that it׳s better to use this training method besides the routine training of nurses for MI patients.

## Specifications Table

**Table:** 

Subject area	*Medicine, Clinical Research, Nursing*
More specific subject area	*Myocardial Infarction management and nursing*
Type of data	*Table, Text file, Figure*
How data was acquired	*Researcher-made and validated questionnaire analysis*
Data format	*Raw, analyzed, descriptive and statistical data*
Experimental factors	*Sample consisted of 70 patients with acute MI, who were randomly divided into two groups of intervention (PE) and control groups (data are presented in the*Fig. 1*).*
	*After inviting the patients, the researcher-made questionnaire including demographic data and questions which were related to the effects of peer-education on quality of nursing were completed.*
	*The cardiac self-efficacy rate in two groups were investigated by filling out the cardiac self-efficacy questionnaire.*
	*The data was collected at the baseline and then after four weeks of the intervention.*
Experimental features	– *Data was gathered using a randomized control trial (RCT) design.* – *Data suggests that PE is one of the most important factors that can affect the self-efficacy in patients with MI*
Data source location	*Zabol, Iran*
Data accessibility	*Data is included in this article*
Related research article	*Esmaeili R, Jannati Y, Ghafari R, Charati JY, Jelodar H (2017). A clinical trial comparing the effect of peer education and orientation program on the anxiety levels of pre-CABG surgery patients. J Med Life, 8(2).*

## Value of the data

• The data can be used by clinicians and health policy makers in order to provide a better care for MI patients.
• The data provides information about the effect of PE on the self-efficacy in patients with MI which is important in management of MI.
• Our data showed that implication of PE for MI patients may resulted in a higher self-efficacy compared to those who were received routine nursing education.
• The data suggests that PE can be useful for providing a better care in MI patients, thus, it is recommended that it׳s better to use this training method besides the training of nurses for MI patients.

## 1 Data

MI is the most common cardiovascular disease [1]. Sol et al. [2] reported that patients with cardiovascular disease can effectively control and manage the signs and symptoms of their disease by improving self-efficacy [2]. Also, educating and improving self-efficacy of patients with MI is important to take care of them [3].

Table 1 represents basic characteristics of patients with MI in two groups of PE and control. According to the Table 1, most patients were 51–60 age group (*P *= .26), and majority of the participants were male with no difference between two groups (*P* = .78). According to the data of the present article, 66.7% of intervention group and 46.7% of control group had lower education (*P* = .06) and the rate of smoking was equal among two groups (60%).

**Table 1:** Demographic profile of patients with myocardial infarction in two groups of peer education and control.

Variable		Group		*P*-value
		Peer education (*n* = 30)	Control (*n* = 30)	
		Number (%) or Mean (Standard deviation	Number (%) or Mean (Standard deviation	
Age	41–50	7(38.9)	11(61.1)	.260^b^
	51–60	23(54.8)	19(45.2)	
				
Sex	Male	19(48.7)	20(51.3)	.787^b^
	Female	11(52.4)	10(47.6)	
				
Marital status	Married	28 (40)	27 (49.1)	1.00^b^
	Single	2(40)	3 (59.3)	
				
Employment status	Employee	3 (10)	0 (0)	.246^c^
	Self-employed	18 (60)	22 (73.3)	
	Housekeeper	9 (30)	8 (26.7)	
				
Residency	City	18(45)	22(55)	.273^b^
	Village	12(60)	8(40)	
				
Level of Education	Illiterate	11 (40.7)	16 (59.3)	.182^b^
	Primary	8 (80)	2 (20)	
	Diploma	10 (50)	10 (50)	
	higher diploma	1(33.3)	2(66.7)	
				
Tobacco consumption	Smoking	12 (40)	12 (40)	1.000^b^
	Non-smoking	18 (60)	18 (60)	
Body mass index		26.36 (2.66)	25.46 (4.99)	.512^a^

a Independent t-test.

b Chi-square test.

c Fisher׳s Exact test.

Table 2 shows that there is no statistically significant difference in means (± SD) of self-efficacy between the PE and control group before the intervention. According to the Table 3, our data suggests a significant difference in mean (± SD) of self-efficacy between pre- and post-intervention among intervention group (*P* = .001) and this association was not found for control group (*P* = .30).

**Table 2:** Mean (± SD) of self-efficacy score in two groups of peer education and control before intervention.

Groups	Peer education (*n* = 30)	Control (*n* = 30)	*P*-Value
	Mean (± SD)	Mean (± SD)	
Three days after myocardial infarction (pre-intervention)	30.70 (13.41)	39.16 (21.02)	.069^a^

a Independent t-test.

**Table 3:** Mean and standard deviation of self-efficacy score in two groups of peer education and control before and after intervention.

Groups		Mean (± SD)	*t*	*P* value
Peer education (*n* = 30)	Pre-intervention	30.70 (± 13.41)	− 3.76	.001
	Post-intervention	42.96 (± 9.34)		
				
Control (*n* = 30)	Pre-intervention	39.16 (±21.02)	− 1.04	.304
	Post-intervention	45.93 (±17.26)		

## 2 Experimental design, materials, and methods

### 2.1 Study area description

The data was gathered at the Zabol teaching hospital, affiliated with the Zabol University of medical sciences in Zabol, Sistan and Baluchestan Province, Iran. The canter is a principle referral center for all disease including hurt disease in this province [4]. This province is located in the Southeast of the country, neighboring two countries including Pakistan and Afghanistan. The province is one of the largest one in country, with an area of 181,785 km^2^ and its population is estimated to be around 2.5 million. Zabol county has around 130,600 populations and it is located near Lake Hamun [5]. Fig. 2 Illustrates the overview of the city. Zabol teaching hospital with 422 beds is located in the centre of Sistan and Baluchestan in the Southeast of Iran.

**Fig. 1:**
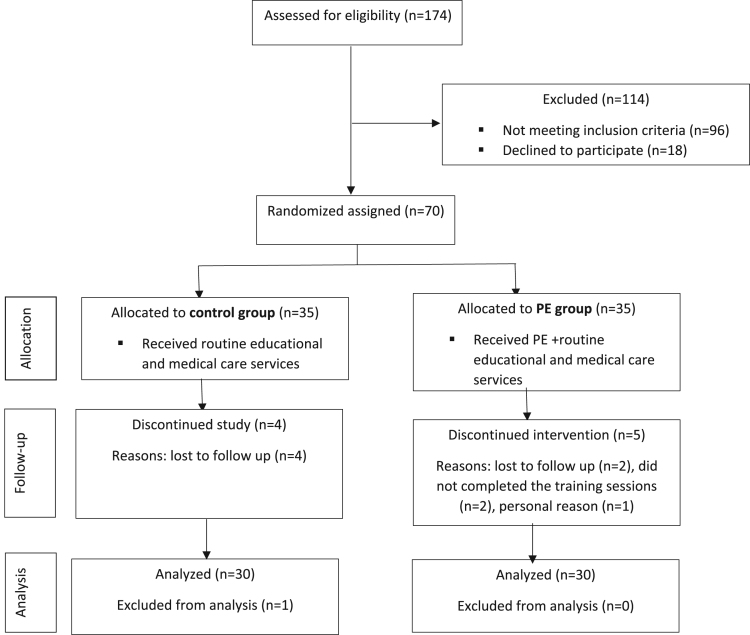
Study flow diagram.

**Fig. 2:**
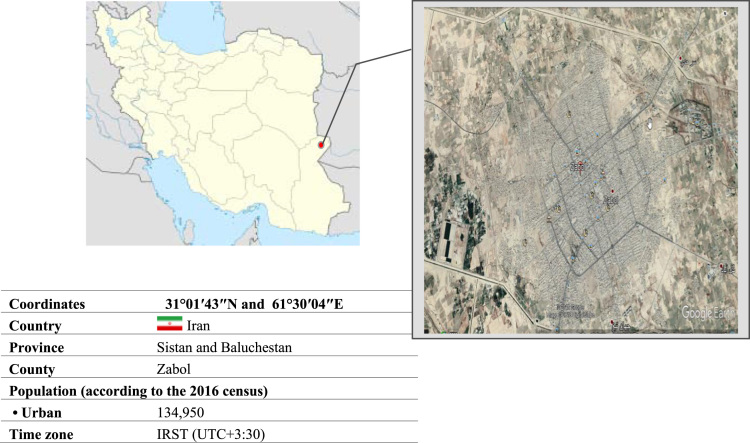
Location of the study area Zabol Hospital, in the Southeastern of Iran.

### 2.2 Experimental design, materials and methods

Zabol Medical University approved and registered the study protocol (IRCT code: IRCT20160050225237N2, and ethical approval number: zbmu.1.REC.1394.136).

As shown in Fig. 1 in total we assessed 174 patients for eligibility, of them 96 patients did not meet our inclusion criteria and 18 declined to participate. Finally, we include 70 patients with MI that randomly assigned to PE group (*n* = 35) and control group (*n* = 35). We used two tools for data collection. Patients’ demographic data questionnaire, consisting of two parts: the first section had questions about general details such as age, education, marital status, while the second section had questions about health status information—these were set after literature evaluation and consultation with professors who were expert in cardiovascular diseases. Cardiac self-efficacy questionnaire was the second questionnaire. The questionnaire Reliability was confirmed with coefficient Alfa 0.91. Then, the data of 30 intervention and 30 control individuals were analyzed using SPSS version 24. The data were analyzed, applying descriptive and statistical tests including independent t-test and pair t-test. *P* value < .05 was considered to be statistically significant.
