# Supplementary material for: Data based investigation of the peer education methods on self-efficacy in patients with myocardial infarction using a randomized control trial design
Source: Data Brief. 2018 Sep 5;20:1347–52. doi: 10.1016/j.dib.2018.08.190 (PMC6146448; doi:10.1016/j.dib.2018.08.190)
Supplement: Supplementary file 1 — Supplementary material [file mmc1.docx]

**Declaration of interest**

**Conflict of Interest:**

**There is no conflict of interest to declare**

**Funding:**

**Zabol University of Medical Sciences (no: 1394.136)**
